# Phylogenetic relationships among tribes of the green lacewing subfamily Chrysopinae recovered based on mitochondrial phylogenomics

**DOI:** 10.1038/s41598-017-07431-1

**Published:** 2017-08-03

**Authors:** Yunlan Jiang, Ivonne J. Garzón-Orduña, Shaun L. Winterton, Fan Yang, Xingyue Liu

**Affiliations:** 1 Department of Entomology, China Agricultural University, Beijing, 100193 China; 2 California State Collection of Arthropods, California Department of Food and Agriculture, Sacramento, CA 95832 USA

## Abstract

Chrysopidae (green lacewings) is the second largest family in Neuroptera, and it includes medium-size lacewings largely recognized by the presence of golden-colored eyes, bright green bodies and delicate wings with dense venation patterns. The subfamily Chrysopinae includes 97% of the species diversity in the family and it is currently divided into four tribes: Ankylopterygini, Belonopterygini, Chrysopini and Leucochrysini. Here we sequenced and annotated the nearly complete mitochondrial genomes of four species of each these tribes: *Abachrysa eureka*, *Italochrysa insignis*, *Leucochrysa pretiosa*, *Parankyloteryx* sp. We then reconstructed the phylogenetic relationships with estimated divergence times among tribes of Chrysopinae based on the mt genomic data. Our results suggest that Chrysopinae sans *Nothancyla verreauxi* evolved as two reciprocally monophyletic lineages formed by stem members of the tribes Leucochrysini plus Belonopterygini on one hand, and the stem members of Ankylopterygini plus Chrysopini on the other. Our estimations of divergence times place the diversification of stem Chrysopinae into the extant tribes during the Middle Jurassic to Late Cretaceous. The relatively young ages previously estimated for the green lacewing divergences were probably underestimated due to false inferences of homology between non-sister taxa that are later correctly identified as homoplasy after more taxa are added.

## Introduction

Green lacewings (Chrysopidae) are charismatic, medium-sized neuropterans frequently encountered in most ecosystems. They can be typically recognized from other lacewings by the highly modified wing venation, delicate appearance and bright green coloration^1^. Chrysopidae is second only to Myrmeleontidae in species richness comprising over 1400 species distributed worldwide, some of which are important beneficial organisms used in the integrated biological control^2^. The extant members of the family are divided into three extant subfamilies: Chrysopinae, Apochrysinae, and Nothochrysinae. Chrysopinae is by far the largest subfamily in species number, including 97% of all chrysopid species, and is subdivided into four tribes, Ankylopterygini, Belonopterygini, Chrysopini and Leucochrysini^1^. Of these, the tribe Chrysopini contains the vast majority of the genera and species. This classification was established as a result of the comprehensive generic revision of the family conducted by Brooks and Barnard^1^, who relied greatly on wing venation and genital features. The significance of the chrysopid revision made by Brooks and Barnard is unquestionable since it served as a template for systematics studies on the family for over two decades^1^. However, the absence of a resolved phylogenetic hypothesis was a distinct limitation, and over the years several attempts were made to elucidate the relationships within Chrysopidae, while at the same time testing the monophyly of suprageneric groups suggested by Brooks and Barnard^1^. With this aim, studies have resorted to the use of adult morphology^1, 3^, mitochondrial fragments COI and 16S^4^, several nuclear fragments^5, 6^ and more recently to the use of complete mitogenomes^7, 8^. Mitogenomes became popular in insect phylogenetics as a result of the development of cheaper and more efficient sequencing techniques, and in the last two decades the annotation of mitogenomes for non-model organisms has increased at a steady pace. In the case of Neuroptera, sequencing and annotation of mitogenomes began almost 10 years ago^9^ and today there are 40 published mitogenomes; this achievement recently allowed the formulation of the first hypothesis for Neuropterida based exclusively on the phylogenetic signal provided by mitogenomes^8^. Likewise within Chrysopidae, mitogenomes have proven to be informative, as they helped place at last the enigmatic monotypic genus *Nothancyla verreauxi*, whose phylogenetic affinities to the other subfamilies of Chrysopidae remained ambiguous for many years^7^.

So far the phylogenetic hypotheses proposed for Chrysopidae vary greatly among authors; this is particularly true for the relationships among the tribes of Chrysopinae, which remain largely equivocal. Persistent questions regarding tribal relationships within Chrysopinae include the possible paraphyly of Chrysopini^5, 6^, the generic membership and delimitation of Leucochrysini and Belonopterygini^10, 11^ and the position of *Nothancyla* Navás within Chrysopinae^4, 7, 12^.

In this study, we provide the results of sequencing and annotating the mitochondrial genomes of four species of the subfamily Chrysopinae. These species each belong to Ankylopterygini, Belonopterygini and Leucochrysini, chrysopinae tribes whose mt genomes have not been sequenced previously. We use these and the mitochondrial genomes previously sequenced for five species of Chrysopidae^7, 13, 14^ to elucidate the higher-level phylogeny and estimation of divergence times among chrysopine tribes of Chrysopinae.

## Results and Discussion

### Gene structure

**Figure 1:**
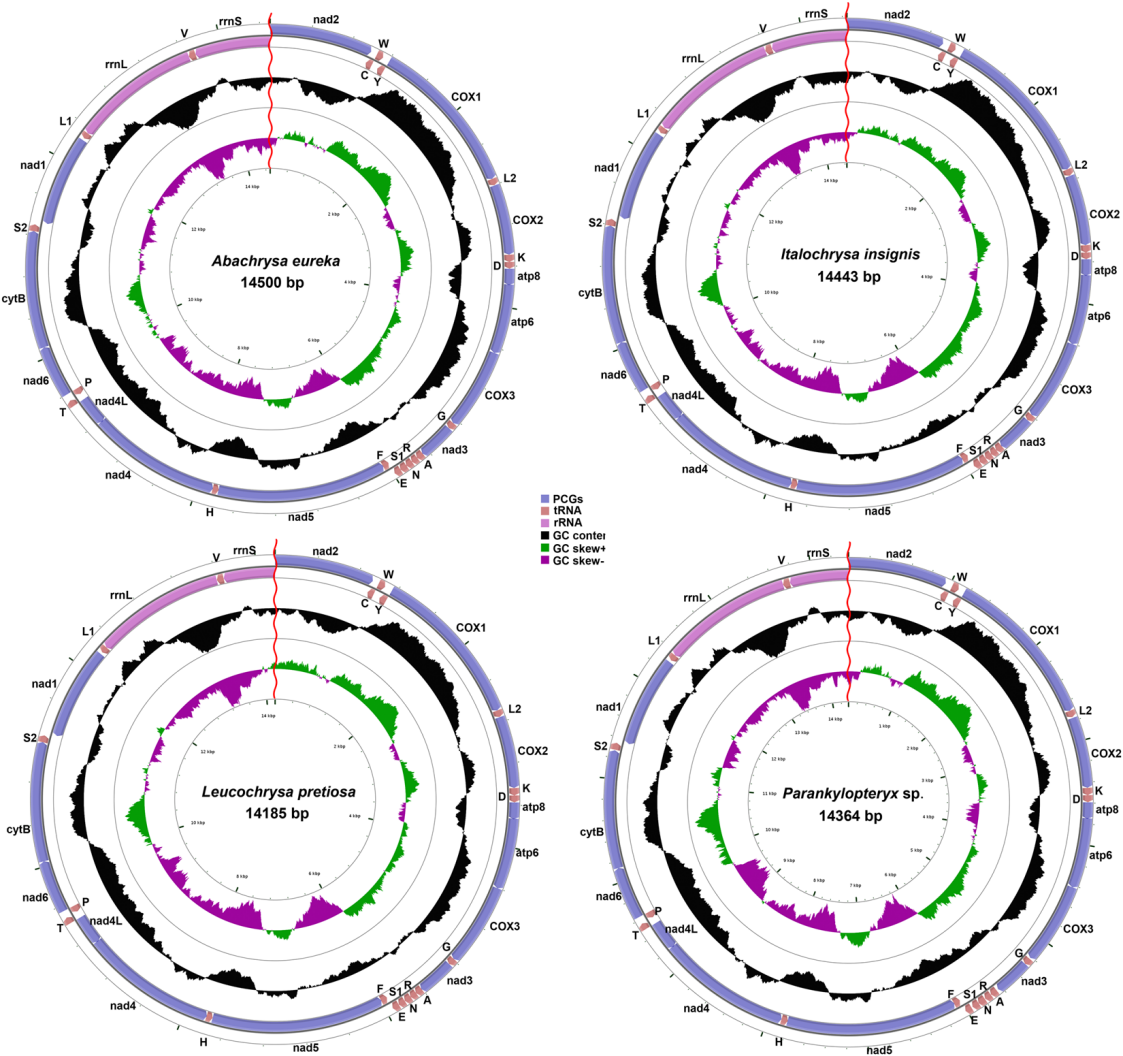
Mitochondrial map of the four species we sequenced. Circular maps were drawn with CGView^45^. The arrows indicated the orientation of gene transcription. The tRNAs are denoted by the color blocks and are labelled according to the IUPACIUB single-letter amino acid codes (L1: UUR; L2: CNU; S1: AGN; S2: UCN). The GC content was plotted using a black sliding window, as the deviation from the average GC content of the entire sequence. GC-skew was plotted as the deviation from the average GC-skew of the entire sequence. The inner cycle indicated the location of the genes in the mt genome.

The sequence of *Abachrysa eureka* (Banks, 1931) (Belonopterygini) is 14500 bp in size, containing genes, with 13 PCGs, 19 tRNAs and 2 rRNAs (Fig. 1), while that of *Italochrysa insignis* (Walker, 1853) (Belonopterygini) is 14433 bp in length consisting the same 33 complete genes and partial sequence of *nad2*. The sequences of *Leucochrysa* (*Leucochrysa*) *pretiosa* (Banks, 1910) (Leucochrysini) and *Parankylopteryx* sp. (Ankylopterygini) were 14185 bp and 14364 bp respectively, containing the same 32 complete genes with *A*. *eureka* and partial sequences of *nad2* and *rrnS*. Because of the high A + T composition and complicated secondary structure, we were unable to amplify the control regions as well as the genes approaching it in the four species. Gene orders of all species sequenced are identical and as a group, they all differ from the ancestral insect mt genome arrangement^15^, at the translated positions of *trnC* and *trnW*, which are translocated. As shown previously by multiple studies, this translocation is universal and exclusive to Neuroptera^7, 8, 13, 16–18^, the exceptions are Coniopterygidae, Nevrorthidae, Sisyridae and Osmylidae, whose mt genomes exhibit the ancestral arrangement with no translocation of *trnC* and *trnW* ^8, 19^.

There are overlaps between every two genes in the mt genomes we sequenced. Two gene pairs including *atp8*-*atp6* and *nad4*-*nad4L* overlap 7 nucleotides, also sharing the sequence ATGNTAA which has been reported in many other insect mt genomes with difference appeared as ATGATAA and ATGTTAA respectively^20, 21^. In addition, there are small non-coding intergenic spacers upstream and downstream of *nad1* about 20–30 bp in length.

### Comparison of tRNA structure

**Figure 2:**
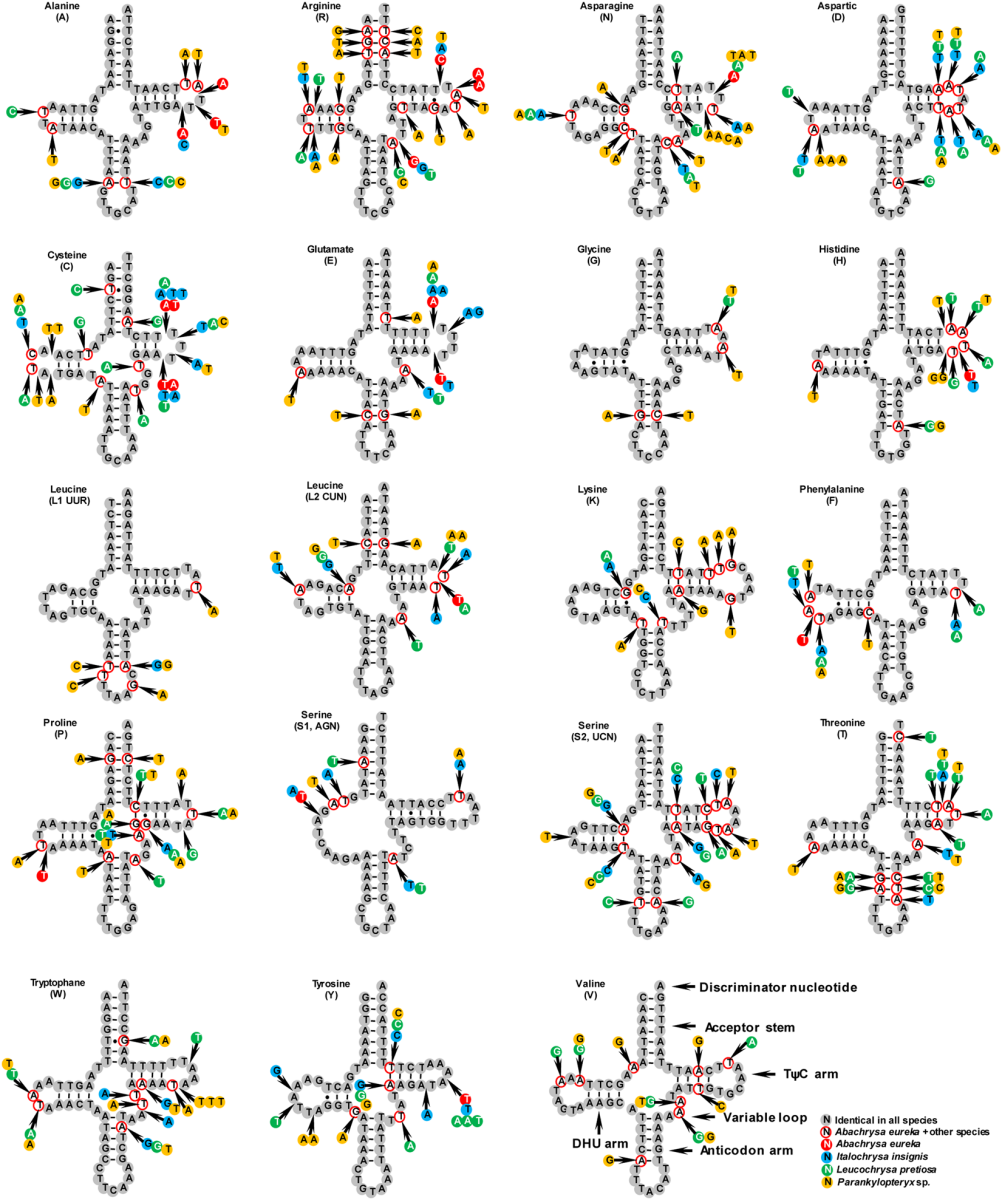
Inferred secondary structure of 19 tRNAs in the mt genome. Most tRNAs are labeled with the abbreviations of their corresponding amino acids. Dash (-) indicates Watson-Crick bonds and dot (•) indicates GU bonds.

There are 19 tRNAs found in the partial mt genome sequences of the four species sequenced here. The length of the tRNAs ranged from 62 to 72 bp. Twelve genes are located on the J-strand and the other seven are located on the N-strand. Most of the tRNAs can be folded into the typical clover-leaf structure (Fig. 2) except *trnS1* (*AGN*), with its dihydorouridine (DHU) arm forming a simple loop, which is a common phenomenon in sequenced Neuropterida mt genomes^7, 22^. The aminoacyl (AA) stem (7 bp) and the anticodon loop (7 bp) are conservative in length. The length of the anticodon (AC) stems is constantly 5 bp, with exception of *trnK*, where a mismatch is present in the first pair of their anticodons. The DHU and TΨC (T) stems are really variable in the loops and stems (Fig. 2). Based on the secondary structure models, mismatched base pairs (i.e. U-U and A-C pairs) were found in the tRNAs. Nucleotide substitutions rarely occur on acceptor and anticodon stems. The changes are fully compensatory base changes (cbcs) (e.g. G-C vs A-T on the acceptor stem of *trnR* and *trnP*) or hemi-cbc (e.g. G-T vs A-T on the acceptor stem of *trnT* and *trnW*)^23^, which are more restricted than that on TΨC (T) and DHU loops. In addition, substitutions involving two full cbcs are found in the *trnT* anticodon stem and the *trnR* acceptor stem.

In previous studies of insect phylogeny, tRNAs have rarely been used. However, the presence of full cbcs and hemi-cbcs may characterize taxa at different taxonomic levels, as well as the secondary structure of tRNAs^24^. The base substitution in stems and loops may provide some evidence in reference to the phylogeny. Sequences of the variable tRNAs of *Parankylopteryx* sp. (Ankylopterygini) are quite different from that in the other three species we sequenced (Fig. 2), e.g. *trnR* and *trnS2* (*UCN*). In addition, among these variable tRNAs, the acceptor stems of *trnL2* (*CUN*) and *trnP* possess T-A pair in *Parankylopteryx* sp., while in the other taxa the C-G pair is present instead. The sequences of tRNAs in *Abachrysa eureka* (Belonopterygini) and *Italochrysa insignis* (Belonopterygini) appear to be invariable, and even identical in *trnG* and *trnV*. The sequences of tRNAs in *Leucochrysa pretiosa* (Leucochrysini) are more similar to that of *Abachrysa eureka* than *Parankylopteryx* sp. (see Fig. 2). This supports the result of phylogenetic analysis that Belonopterygini is the sister group of Leucochrysini, while Ankylopterygini is distantly related to them.

### Comparison of *rrnL* secondary structures

**Figure 3:**
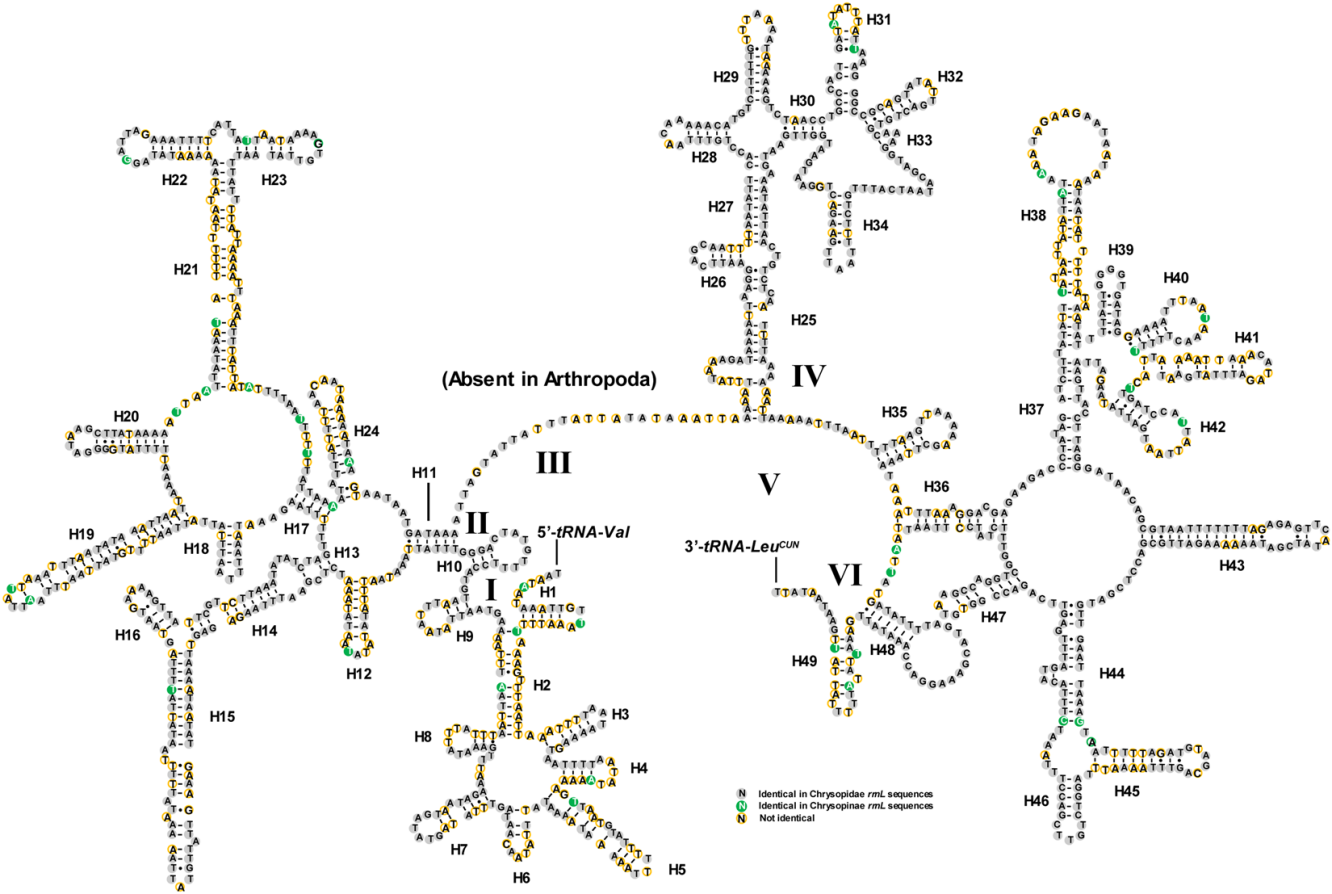
Predicted secondary structure of the *rrnL* in the *Abachrysa eureka* mt genomes. Roman numerals denote the conserved domain structure. Dash (-) indicates Watson-Crick base pairing and dot (•) indicates G-U base pairing.

The lengths of *rrnL* of the four green lacewings we sequenced were determined from 1301 bp to 1318 bp (see bioinformatics analysis). The inferred secondary structure model for the *rrnL* of *A*. *eureka* is provided in the Fig. 3. The structure of *rrnL* largely resembles previously published structures for *Libelloides macaronius* ^17^ and *Nothancyla verreauxi* Navás^7^. The secondary structure consists of five canonical domains (I-II, IV-V) with domain III absent, which is a typical trait in arthropods^25^ (Fig. 3), including 50 helices. The predicted secondary structure of domain I includes eight helices and is consistent among all nine chrysopid taxa. There are conserved sites unevenly distributed throughout the *rrnL* secondary structure. For example, the sequence of *rrnL* of *A*. *eureka* is in a visible uniformity to 69.01% with the multiple alignments of *rrnL* in Chrysopidae and 71.84% in Chrysopinae. The region with highest level of invariable positions is located in domain IV, especially the triangle region formed by helices H30 to H34, while that with lowest level of invariable positions is observed respectively in domains I-II.

### Phylogenetic relationships among tribes of Chrysopinae

**Figure 4:**
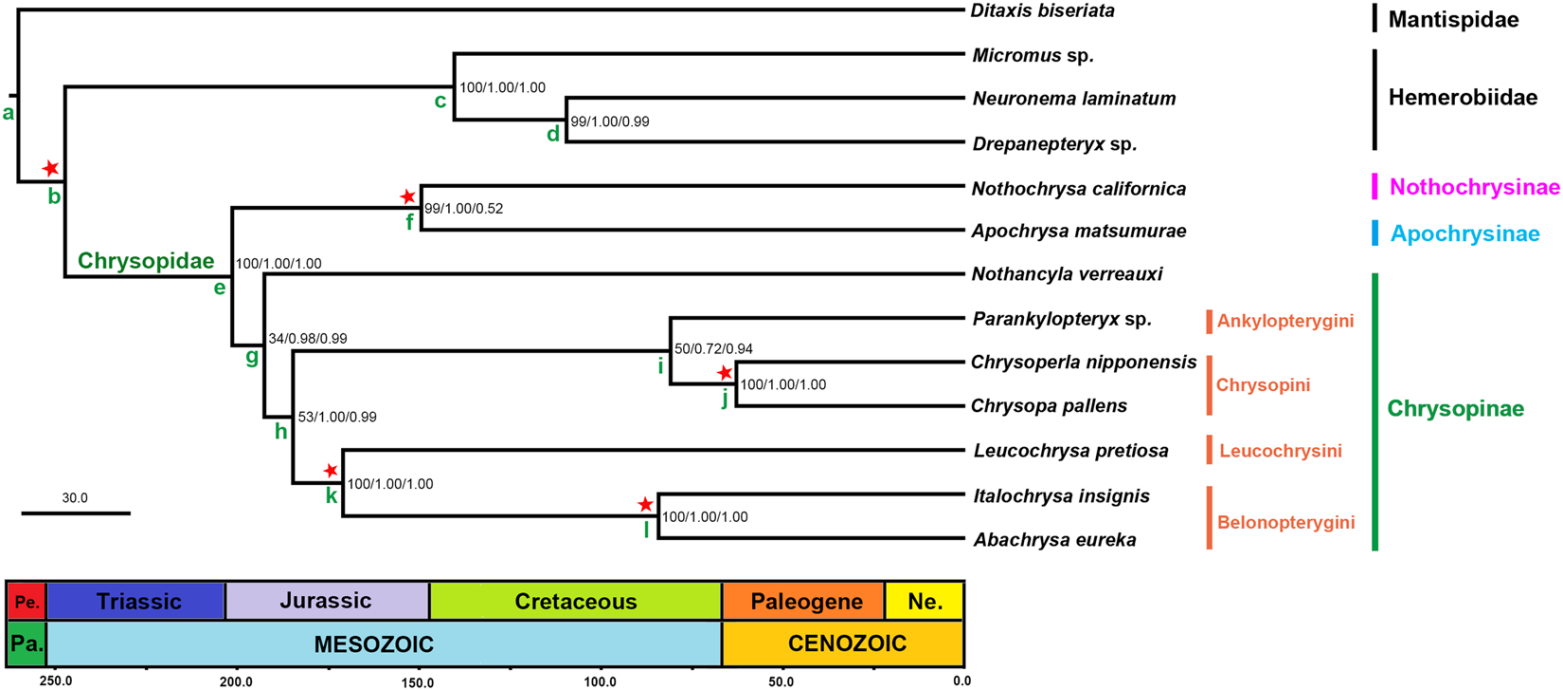
Phylogenetic hypothesis of green-lacewings obtained with Bayesian inference and maximum likelihood based on mitogenomes, divergence time estimates calculated with Phylobayes are also featured. Branch support values are featured at their respective node in the following order: bootstrap values/posterior probabilities (resulted under partition-specific models)/posterior probabilities (resulted under CAT model). Letters on nodes correspond to the age estimates featured in Table 2. Scale units are in millions of years. Stars indicate the node with fossil calibration and letters are used as arbitrary labels.

Bayesian and maximum likelihood analyses generated phylogenetic trees with the same topology as the chronogram in Fig. 4. The sampling list of the present phylogenomic analysis is shown in Table 1. Accordingly, Chrysopinae was recovered sister to Apochrysinae plus Nothochrysinae, which is consistent with results of previous studies using nuclear genes only^5, 6^. The sister relationship between Nothochrysinae and Apochrysinae recovered here had a high bootstrap value and strong posterior probability support resulted under partition-specific models, but with rather low posterior probability support resulted under CAT model, which indicates instability concerning the relationships between these subfamilies. A previous mitogenomic analysis for the family^7^ found Apochrysinae sister to the remaining Chrysopidae, a result that agrees partially with hypotheses based on morphology^1, 26^. Taken together these results suggest that the signal provided by mitogenomes seems equivocal and unable to resolve relationships in the deepest part of the Chrysopidae tree.

**Table 1:** Sampling list of the present phylogenomic analysis.

Family	Species	GenBank accession number	Reference
Chrysopidae	*Abachrysa eureka*	KY587199	Present study
	*Italochrysa insignis*	KY587200	Present study
	*Leucochrysa pretiosa*	KY587201	Present study
	*Parankylopteryx* sp.	KY587202	Present study
	*Apochrysa matsumurae*	AP011624	Haruyama *et al*.^13^
	*Chrysopa pallens*	JX033119	He *et al*.^14^
	*Chrysoperla nipponensis*	AP011623	Haruyama *et al*.^13^
	*Nothancyla verreauxi*	KP264629	Dai *et al*.^7^
	*Nothochrysa californica*	KP264630	Dai *et al*.^7^
Hemerobiidae	*Drepanepteryx phaleonoides*	KT425075	Wang *et al*.^8^
	*Neuronema laminatum*	KR078257	Zhao *et al*.^35^
	*Micromus* sp.	KT425087	Wang *et al*.^8^
Mantispidae	*Ditaxis biseriata*	FJ859906	Cameron *et al*.^16^

As a genus that exhibits characteristics of both Apochrysinae and Chrysopinae, *Nothancyla* was recovered here as the sister group to all other Chrysopinae, in agreement with the result from a previous study using fewer mt genomes^7^. The remaining Chrysopinae are grouped into two clades, Leucochrysini + Belonopterygini and Ankylopterygini + Chrysopini, which is in accordance with the relationships proposed by Brooks^3^. Leucochrysini and Belonopterygini share certain synapomorphic genitalic characters suggesting a closer relationship between the two tribes. For example, the gonarcus is broad, transverse and hardly arcuate with gonocornua, and the arcessus is short and broad with a strong hook and lateral membranous lobes at the apex in both tribes. Nevertheless, Belonopterygini is characterized by the long and broad intramedian cell, which is usually quadrangular. The synapomorphy supporting the sister group relationship between Ankylopterygini and Chrysopini is the narrow arcessus. Ankylopterygini genera can be distinguished by the narrow hind wings (length:breadth > 3.3:1)^1^, densely setose forewings with broad costal region, scythe-like mandibles and apically constricted maxillary palps.

Remarkably, the relationships among tribes of Chrysopinae in this study are different from other results from DNA data with weak support in various topologies. Chrysopini was recovered as sister to the remaining Chrysopinae the other three tribes in the study by Winterton and de Freitas^4^, with Ankylopteryigini sister to Leucochrysini. Moreover, subsequent studies using different molecular datasets did not support the monophyly of Chrysopini^5, 6^. A recurring issue with molecular phylogenetic studies of Chrysopidae is either limited character sampling^13^ or limited taxon sampling^4, 7^. This study again presents large amount of DNA sequence data, but also begins to build upon the limited taxon sampling of previous studies to include representatives of all major chrysopid lineages. The next step is to expand on this taxon sampling considerably, to begin testing the monophyly of these lineages in a phylogenetic context.

The larval stages of chrysopids exhibit the unusual behavior is entangling debris in elongate, recurved setae on their dorsum to form a trash packet. This is used for camouflage and as a physical shield against predators and parasites^27^. However, not all green lacewing larva carry a trash packet and those that do not (so called ‘naked’ larvae) display different behaviors (i.e. generally nocturnal, faster moving). Trash carrying is not found in larvae of the closely related Hemerobiidae. While *Nothochrysa* larvae do carry a trash packet, most Nothochrysinae do not. It is found in most representative sampled in Chrysopinae, except *Chrysoperla* and *Chrysopa*. These results suggest two evolutionary scenarios that (1) larval trash carrying evolved once in stem chrysopids and then has been lost at least twice, in both Nothochrysinae and in at least some Chrysopini; and (2) it evolved twice in stem Chrysopinae and within Nothochrysinae and then has been lost once in at least some Chrysopini.

### Divergence time estimates

The chronogram with divergences times estimations obtained with PhyloBayes using fossil calibrations is featured in Fig. 4. Mean age values and 95% high posterior density (HPD) intervals for each node are presented in Table 2. The estimated age at the node on the chronogram represents a mean of the probability distribution of ages for that node, with time intervals within 95% probability of the mean age. Estimations of divergence times based on the concatenation of the 13 PCGs and rRNAs placed the divergence between stem Nothochrysinae + Apochrysinae from stem Chrysopinae in the Late Triassic to the Early Jurassic (~201 MA). In addition, stem Apochrysinae diverged from stem Nothochrysinae at ~149 MA, in the end of Jurassic to onset of Cretaceous. While the first split within Chrysopinae was that stem *Nothancyla verreauxi* diverged from stem Chrysopinae around 193 MA. The diversification of Chrysopinae into two reciprocally monophyletic lineages occurred approximately 185 MA. The tribes Leucochrysini was estimated to be the oldest of the four tribes, diverging from Belonopterygini in the Middle Jurassic (~171 MA), in contrast to Ankylopterygini and Chrysopini which diverged from their common ancestor close to the end of Cretaceous at ~81 MA.

**Table 2:** Mean divergence times and 95% high posterior density (HPD) intervals for each node of the topology presented in Fig. 4.

Node	Estimations using fossil calibrations			Estimations using fixed COI substitution rate		
	Mean age	Inferior 95%	Superior 95%	Mean age	Inferior 95%	Superior 95%
a	260.07	258.13	262.02	7.13	4.29	10.77
b	247.24	222.15	259.66	7.02	4.24	10.64
c	140.32	62.86	211.35	5.00	3.17	7.67
d	109.51	41.48	189.51	4.45	2.71	6.71
e	201.33	181.00	227.06	3.34	2.18	5.47
f	149.38	34.56	207.49	3.07	1.81	4.61
g	192.51	175.27	217.07	3.52	2.05	5.19
h	184.65	170.49	207.39	3.32	2.04	5.01
i	80.92	26.25	181.76	3.06	1.82	4.56
j	62.86	14.62	174.40	2.10	1.21	3.14
k	170.91	163.20	190.88	2.74	1.68	4.12
l	84.22	21.01	156.46	2.04	1.24	3.11

Time-scale units are in millions of years.

When comparing the divergence times estimations from this study with those from that of Dai *et al*.^7^ (based also on mitogenomes but with a smaller taxonomic sampling), we found that the divergence time estimates obtained here are significantly older than those obtained previously. This surprising result (given the similarities in the sourced data and calibrations used by both studies) is presumably due to the “node density effect”^28^ on branch lengths, which results from the detection of homoplasy, initially mistaken as homology, once more taxa is included. Homoplasy erroneously inferred as synapomorphies when less taxa are included, will make unrelated taxa look more similar and (under a clock) more recently diverged than they are.

As deep phylogenetic divergences were included in this study, theoretically given the known rapid nucleotide substitutions of mt DNA, a strict molecular clock model failed (or underestimate) at accounting for the whole extend of the sequence divergence because of saturation (aka. Multiple hits). The use of a strict molecular clock model does not leave room to express uncertainty around the estimations, as it provides direct measurements resulted from the application of the rate. In addition, previous research suggested that there is noticeable rate variation among lineages so that the use of a strict molecular clock model could not account for such rate variation^8^. As a result of this, the divergence time estimated using a strict molecular clock model was too young to be believable (see Table 2 for mean age values and 95% HPD intervals for each node).

The fact that branch lengths estimations are affected by taxon sampling is a problem for relaxed molecular clocks that has been reported multiple times^29–31^. Therefore, this means that as old as the ages obtained here with a larger taxonomic sample are, they represent a better hypothesis than the one obtained by Dai *et al*.^7^. Of course all things being equal (e.g. calibrations), we would expect future divergence times estimations for Chrysopidae to be even older if they are estimated for a more inclusive taxon sampling.

## Materials and Methods

### Specimens and DNA extraction

Specimens from four species belonging to the Chrysopinae tribes: Belonopterygini Navás, 1913, Leucochrysini Adams, 1978, and Ankylopterygini Navás, 1910, were collected at different fieldwork expeditions. The specimen of *A*. *eureka* was collected on June 2, 2002, in Briarwood Preserve, Bienville Parish, Louisiana, U.S.A. by A. J. Ames. The specimen of *L*. (*L*.) *pretiosa* was collected on May 8–15, 2009, in Bagua/Tarapoto Rd (5 N) at Km 403, Distrito Aguas Verdes, Dept Amazonas, PERU by M. E. Irwin and G. Antón Amaya. The specimen of *I*. *insignis* was collected during March 13 to April 8, 2008, in Warrumbungle National Park, New South Wales, Australia by S. L. Winterton, J. S. Bartlett and D. J. Tree. The specimen of *Parankylopteryx* sp. was collected during April 21 to May 1st, 2014 by S. Gaimari and M. Hauser in Kakum National Park, Central Region nr. Abroto, Ghana. After capture, all the specimens were preserved in 95% ethanol and stored at −20 °C in the Entomological Museum of China Agricultural University (CAU) previous to the DNA extraction. Total genomic DNA was extracted from thoracic muscle tissue using the TIANamp Genomic DNA Kit (Tiangen Biotech, Beijing, China).

### PCR amplification and sequencing

The mt genome sequences were generated by amplification of overlapping PCR fragments. Primers for the present PCR are provided in Table S1–4. All PCRs used NEB Long Taq DNA polymerase (New English BioLabs, Ipswich, MA) under the following amplification conditions: 30 s at 95 °C, 40 cycles of 10 s at 95 °C, 50 s at 43–56 °C, 1 kb/min at 65 °C depending on the size of amplificons, and the final elongation step at 65 °C for 10 min. The quality of PCR products was evaluated by 1% agarose gel electrophoresis. All PCR products were sequenced in both directions using the BigDye Terminator Sequencing Kit (Applied Bio Systems) and the ABI 3730XL Genetic Analyzer (PE Applied Biosystems, San Francisco, California USA) with aforementioned primers for primer walking.

### Bioinformatic analysis

The nearly complete mt genome sequences of *A*. *eureka*, *I*. *insignis*, *L*. (*L*.) *pretiosa* and *Parankylopteryx* sp. were deposited in GenBank with accession numbers KY587199, KY587200, KY587201 and KY587202, respectively. Sequences assembly was done by using ContigExpress. tRNAs were identified by tRNAscan-SE Search Server v.1.21^32^. The secondary structure of the tRNAs were generated by tRNAscan-SE as stated, while some of them were predicted by the relevant genes from green lacewing species *Nothancyla verreauxi* Navás and *Nothochrysa californica* Banks^7^ which could not be generated by tRNAscan-SE. The PCGs were identified as open reading frames corresponding to the 13 PCGs in metazoan mt genomes, whose boundaries were identified by comparing the boundaries of the same PCGs in related neuropteran mt genomes with MEGA 5.0^33^. It is impossible to infer the boundaries of rRNAs accurately because there are no start or stop codons in the rRNA genes. Therefore, the rRNAs were assumed to extend to the boundaries of flanking genes^34, 35^ and were confirmed by alignment to relevant genes of other species of Neuroptera (i.e., *Apochrysa matsumurae* Okamoto^13^, *Nothancyla verreauxi* Navás and *Nothochrysa californica* Banks^7^ using MEGA 5.0. The control regions were identified afterwards by the boundary of the rRNAs genes and compared with other insect mt genomes.

### Phylogenetic analysis

Complete or nearly complete mt genomes of nine green lacewings were selected as the ingroup taxa in the phylogenetic analysis, this sampling ensured the inclusion of species from all three subfamilies of Chrysopidae as well as representative species of four tribes in Chrysopinae. Three brown lacewing (Hemerobiidae) species i.e. *Drepanepteryx* sp. (GenBank accession number: KT425087), *Neuronema laminatum* Tjeder^36^ and *Micromus* sp. (GenBank accession number: KT425075), and one mantispid species *Ditaxis biseriata* (Westwood) (GenBank accession number: FJ859906) were selected as outgroups.

Alignment of the sequences of the 13 PCGs was inferred from the amino acid sequences using ClustalW in MEGA 5.0^33^, while the rRNA alignments were conducted by G-blocks Server (http://molevol.cmima.csic.es/castresana/Gblocks_server.html). Then, alignments of individual genes were concatenated without stop codons but including all three codon positions for phylogenetic analysis. Bayesian (BI) and maximum likelihood (ML) analyses were made based on the partitioned datasets with partition-specific models (Table S5–6) estimated by PartitionFinder^37^. The BI inference was performed in MrBayes Version 3.1.2^38^, in which two simultaneous runs of 2,000,000 generations were conducted, and the dataset was sampled every 200 generations with a burn-in of 25%. In the ML algorithms, nodal supporting values were assessed by bootstrap values (BP)^39^ calculated with 10000 replicates from 1000 RAxML runs. Also phylogenies were inferred under the heterogeneous model CAT using PhyloBayes 3.3^40^. Four independent tree searches were run until the likelihood of the sampled trees was stabilized and every two runs had satisfactorily converged (maxdiff less than 0.3).

### Divergence time estimation

Divergence time among the green lacewing subfamilies was estimated in PhyloBayes 3.3^40^. The topology obtained in each of the phylogenetic analysis earlier was constrained using the following five minimum age calibrations, (1) the most recent common ancestor (MRCA) of *Italochrysa* and *Abachrysa* was calibrated with an undescribed specimen from the late Eocene in Baltic Amber to a minimum age of 38 Ma^41^; (2) the split of *Leucochrysa* from *Italochrysa* + *Abachrysa* was calibrated with the mean age of *Leucochrysa* (*Nodita*) *prisca* to a minimum age of 21 Ma^42^; (3) the split of *Chrysoperla* was constrained to a min age of 21 Ma based on *Chrysopa glaesaria* ^42^; (4) the MRCA of Apochrysinae and Nothochrysinae was calibrated with *Adamsochrysa aspera* to a min age of 53 Ma^43^; (5) the MRCA of Hemerobiidae and Chrysopidae was calibrated with a mean age of 163 Ma based on *Mesypochrysa* ^43^. Additionally, a secondary calibration of 260 Ma for the root was placed based on the divergence of Chrysopidae + Hemerobiidae from Mantispidae. In PhyloBayes two chains of autocorrelated lognormal relaxed clock model^44^ were run, using a birth-death process as tree prior for 28 000 cycles with the first 5000 cycles^40^ discarded as burn in.

## Electronic supplementary material

Supplementary information
