# Supplementary material for: Phylogenetic relationships among tribes of the green lacewing subfamily Chrysopinae recovered based on mitochondrial phylogenomics
Source: Sci Rep. 2017 Aug 3;7:7218. doi: 10.1038/s41598-017-07431-1 (PMC5543154; doi:10.1038/s41598-017-07431-1)
Supplement: Supplementary file 1 — Supplementary information [file 41598_2017_7431_MOESM1_ESM.docx]

**Phylogenetic relationships among tribes of the green lacewing subfamily Chrysopinae recovered based on mitochondrial phylogenomics**

Yunlan Jiang^1^, Ivonne J. Garzón-Orduña^2^, Shaun L. Winterton^2^, Fan Yang^1^, and Xingyue Liu^1*^

1. Department of Entomology, China Agricultural University, Beijing 100193, China.

2.California State Collection of Arthropods, California Department of Food and Agriculture, Sacramento, CA 95832, USA.

*Correspondence: Xingyue Liu, Department of Entomology, China Agricultural University, Beijing 100193, China. Email: [xingyue_liu@yahoo.com](mailto:xingyue_liu@yahoo.com.cn)

**Table S1. Primer sequences of *Abachrysa eureka* mt genome used in this study.**

| Number | Primer ID | Nucleotide sequence (5’-3’) | Reference |
| --- | --- | --- | --- |
| 1 | F01(SPA-2756) | ACATTTTTTCCTCAACATTT | Simon *et al.* ^1^ |
|  | R01(SPA-3665) | CCACAAATTTCTGAACACTG | Simon *et al.* ^1^ |
| 2 | F03(SPA-3790) | CATTAAGTGACTGAAAGCAAGTA | Simon *et al.* ^1^ |
|  | R03(SPA-4552) | ATGACCTGCAATTATATTAGC | Simon *et al.* ^1^ |
| 3 | F05(SPA-4792) | GTAGATGCAAGCCCTTGACC | Simon *et al.* ^1^ |
|  | R05(SPA-5731) | ATTGGATCAAATCCACATTC | Simon *et al.* ^1^ |
| 4 | F12(SPB-11335) | CATATTCAACCAGAATGATA | Simon *et al.* ^1^ |
|  | R12(SPB-12067) | AATCGTTCTCCATTTGATTTTGC | Simon *et al.* ^1^ |
| 5 | F14(SPB-12261) | TACCTCATAAGAAATAGTTTGAGC | Simon *et al.* ^1^ |
|  | R14(SPB-13000) | TTACCTTAGGGATAACAGCGTAA | Simon *et al.* ^1^ |
| 6 | F15(SPB-12888) | CCGGTCTGAACTCAGATCATGTA | Simon *et al.* ^1^ |
|  | R15(SPB-13889) | ATTTATTGTACCTTTTGTATCAG | Simon *et al.* ^1^ |
| 7 | F16(SPB-13342) | CCTTTGCACAGTCAAAATACTGC | Simon *et al.* ^1^ |
|  | R16(SPB-14220) | TTATGCACACATCGCCCGTC | Simon *et al.* ^1^ |
| 8 | F17(SPB-14197) | GTAAAYCTACTTTGTTACGACTT | Simon *et al.* ^1^ |
|  | R17(SPB-14745) | GTGCCAGCAAYCGCGGTTATAC | Simon *et al.* ^1^ |
| 9 | F20(SPB-586) | CCATTCCATTTYTGATTTCC | Simon *et al.* ^1^ |
|  | R20(SPB-1738) | TTTATTCGTGGAAATGCTATGTC | Simon *et al.* ^1^ |
| 10 | F22(SPB-2756) | ACATTTTTTCCTCAACATTT | Simon *et al.* ^1^ |
|  | R22(SPB-3389) | TATTCATATCTTCAATATCATTGATG | Simon *et al.* ^1^ |
| 11 | F23(SPC-10621) | CTCATACTGATGAAATTTTGGTTC | Simon *et al.* ^1^ |
|  | R23(SPC-11526) | TTCTACTGGTCGTGCTCCAATTCA | Simon *et al.* ^1^ |
| 12 | TF210 | AATTAAGCTACTAGGTTCATACCC | Simon *et al.* ^1^ |
|  | TR1284 | ACARCTTTGAAGGYTAWTAGTTT | Simon *et al.* ^1^ |
| 13 | TF3399 | ACAATTGGTCAYCAATGATAYTG | Simon *et al.* ^1^ |
|  | TR4061 | GAGAATAAGTTWGTTATCATTTTCA | Simon *et al.* ^1^ |
| 14 | TF5470 | GCAGCTGCYTGATAYTGRCA | Simon *et al.* ^1^ |
|  | TR6384 | TATATTTAGAGYATRAYAYTGAAG | Simon *et al.* ^1^ |
| 15 | TF-J8641 | CCAGAAGAACATAANCCRTG | Simon *et al.* ^1^ |
|  | TR-N9629 | GTTTGTGAGGGWGYTTTRGG | Simon *et al.* ^1^ |
| 16 | TF-J7572 | AAAGGGAATTTGAGCTCTTTTWGT | Simon *et al.* ^1^ |
|  | TR-N8487 | TATCAGSTAATATRGCWGCTCC | Simon *et al.* ^1^ |
| 17 | AF1 | CAGTTTATCACCTAATTTCAGTCAT | Present study |
|  | AR1 | GTTCTATTAATGGAGAAGCTCTATT | Present study |
| 18 | AF2 | ATGGCTGAAAGTAAGTATTGGTCTC | Present study |
|  | AR2 | CCATGGTGTCCTGTAGATCCTAATA | Present study |
| 19 | AF3 | CATTATTAGGATCTACAGGACACCA | Present study |
|  | AR3 | GCTCAAGTGATAGTAATTCCTGAAG | Present study |
| 20 | AF4 | GGATTACATGTATTAATCGGAACAT | Present study |
|  | AR4 | GGTAATAATAAAGCAATTTCTACAT | Present study |
| 21 | AF6 | ATCGATTAATTGATTCTCTTGCTAT | Present study |
|  | AR6 | AAATTAGTAATTACTGTGGCACCTC | Present study |
| 22 | AF7 | CTACATCAAACTGGATCTAATAATC | Present study |
|  | AR7 | TCAAAACATATACTTATTCAAGTTC | Present study |
| 23 | AF8 | GAAACTGGTAAATATCTTTTTCAAC | Present study |
|  | AR8 | CGTAAAGGTCCTAATAAAGTAGGTT | Present study |
| 24 | AF9 | CACAAATTACAATTAATAATCTTCT | Present study |
|  | AR9 | TGAATGGTTGAATGAAATATTAACT | Present study |
| 25 | AF10 | TTTAACTCTCTTAAATTCGAGATTT | Present study |
|  | AR10 | TCTAATTTGTATACCATCGTTATCA | Present study |
| 26 | AF2-1 | AGGAGCTTCTGTTGATTTAGCTATC | Present study |
|  | AR2-1 | CATCAGGATAATCAGAATAACGTCG | Present study |
| 27 | AF2-2 | GCAATTAGTTTCGACCTAATAGTTA | Present study |
|  | AR2-2 | GTGATGATGAAAATTTAATACGATT | Present study |
| 28 | AF2-3 | GATTAAATCATATCTTTGATACCAC | Present study |
|  | AR2-3 | GTTCATTTATGATTACCTAAAGCTC | Present study |
| 29 | AF2-4 | TTAAAACATAAACAATTACACCAGA | Present study |
|  | AR2-4 | TTACTGTGGCACCTCAAAAAGATAT | Present study |
| 30 | AF2-5 | CCTCTAAATAGACTAAAATACCGCC | Present study |
|  | AR2-5 | TATAGATGAAGCTAAAGCTTGCACT | Present study |
| 31 | AF3-1 | TTAAGCCAATAGTTCTAATCATAGG | Present study |
|  | AR3-1 | CCTACACCTGTATCAGCTTTAGTTC | Present study |
| 32 | AF3-2 | AACCTAATGCACCTTCACAAACTCT | Present study |
|  | AR3-2 | TCCTATAGCTAAAGGATGTTTAGTT | Present study |

**Table S2. Primer sequences of *Italochrysa insignis* mt genome used in this study.**

| Number | Primer ID | Nucleotide sequence (5’-3’) | Reference |
| --- | --- | --- | --- |
| 1 | F01(SPA-2756) | ACATTTTTTCCTCAACATTT | Simon *et al*. ^1^ |
|  | R01(SPA-3665) | CCACAAATTTCTGAACACTG | Simon *et al*. ^1^ |
| 2 | F02(SPA-3399) | TCTATTGGTCATCAATGGTACTG | Simon *et al*. ^1^ |
|  | R02(SPA-4061) | GAAAATAAATTTGTTATCATTTTCA | Simon *et al*. ^1^ |
| 3 | F03(SPA-3790) | CATTAAGTGACTGAAAGCAAGTA | Simon *et al*. ^1^ |
|  | R03(SPA-4552) | ATGACCTGCAATTATATTAGC | Simon *et al*. ^1^ |
| 4 | F06(SPA-5747) | CCATTTGAATGTGGRTTTGATCC | Simon *et al*. ^1^ |
|  | R06(SPA-6384) | AAAATTAAAAGCATAATATTGAAG | Simon *et al*. ^1^ |
| 5 | F08(SPA-7077) | TTAAATCCTTTGAGTAAAATCC | Simon *et al*. ^1^ |
|  | R08(SPA-7793) | TTAGGTTGAGATGGTTTAGG | Simon *et al*. ^1^ |
| 6 | F12(SPB-11335) | CATATTCAACCAGAATGATA | Simon *et al*. ^1^ |
|  | R12(SPB-12067) | AATCGTTCTCCATTTGATTTTGC | Simon *et al*. ^1^ |
| 7 | F13(SPB-11876) | CGAGGTAAAGTACCACGTACTCA | Simon *et al*. ^1^ |
|  | R13(SPB-12595) | GTTGGATTTCTAACTTTATTRGARCG | Simon *et al*. ^1^ |
| 8 | F14(SPB-12261) | TACCTCATAAGAAATAGTTTGAGC | Simon *et al*. ^1^ |
|  | R14(SPB-13000) | TTACCTTAGGGATAACAGCGTAA | Simon *et al*. ^1^ |
| 9 | F15(SPB-12888) | CCGGTCTGAACTCAGATCATGTA | Simon *et al*. ^1^ |
|  | R15(SPB-13889) | ATTTATTGTACCTTTTGTATCAG | Simon *et al*. ^1^ |
| 10 | F16(SPB-13342) | CCTTTGCACAGTCAAAATACTGC | Simon *et al*. ^1^ |
|  | R16(SPB-14220) | TTATGCACACATCGCCCGTC | Simon *et al*. ^1^ |
| 11 | F17(SPB-14197) | GTAAAYCTACTTTGTTACGACTT | Simon *et al*. ^1^ |
|  | R17(SPB-14745) | GTGCCAGCAAYCGCGGTTATAC | Simon *et al*. ^1^ |
| 12 | F22(SPB-2756) | ACATTTTTTCCTCAACATTT | Simon *et al*. ^1^ |
|  | R22(SPB-3389) | TATTCATATCTTCAATATCATTGATG | Simon *et al*. ^1^ |
| 13 | F23(SPC-10621) | CTCATACTGATGAAATTTTGGTTC | Simon *et al*. ^1^ |
|  | R23(SPC-11526) | TTCTACTGGTCGTGCTCCAATTCA | Simon *et al*. ^1^ |
| 14 | TF-J7572 | AAAGGGAATTTGAGCTCTTTTWGT | Simon *et al*. ^1^ |
|  | TR-N8487 | TATCAGSTAATATRGCWGCTCC | Simon *et al*. ^1^ |
| 15 | IF1 | TGCCTCCATTTTTAGGATTTTTCCC | Present study |
|  | IR1 | TATGTTCAGCGGGAGGATAATTTTG | Present study |
| 16 | IF2 | AGGTTCTACAGGTCATTATGGTAGA | Present study |
|  | IR2 | ACTCAATTTAATGCACCTTGATTTC | Present study |
| 17 | IF3 | GCATCTAAAAAGTATTGACTTATTC | Present study |
|  | IR3 | GGGGGTATATTTATTAATTCGTTTT | Present study |
| 18 | IF5 | CAGATAATTTTATTCCCGCAAATCC | Present study |
|  | IR5 | TCAAAACATATGCTTATTCAAGCTC | Present study |
| 19 | IF6 | ACTTTATTAGGACCTTTTCGAATTT | Present study |
|  | IR6 | GAATTCAGCAAATTAATATTCACCT | Present study |
| 20 | IF7 | TTTAACTCTCTTAAATTCGAGAAAC | Present study |
|  | IR7 | CTGTTCTGTAAATGATAATCCACGA | Present study |
| 21 | IF2-1 | CTAACCATAAGGATATCGGAACTTT | Present study |
|  | IR2-1 | GCTAATACAACTCCTGTTAAACCTC | Present study |
| 22 | IF2-2 | TTAATGTCTTCACATTTTAATCATC | Present study |
|  | IR2-2 | TTTTTAGATGCAACCTAAATGTTAT | Present study |
| 23 | IF2-3 | GATTAAATCATATCTTTGATACCAC | Present study |
|  | IR2-3 | AAAATGATATTTGACCTCAAGGTAA | Present study |
| 24 | IF4-1 | ATATACTTCGTCTTCTTGTTCGTTC | Present study |
|  | IR4-1 | GTAATGGGCCTTGGTTTATATCTGT | Present study |
| 25 | IFc | GAATATATCTATTACTACCTTAGCA | Present study |
|  | IRc | TTTTTTTATTAGTCATTAAGGGGAT | Present study |
| 26 | IF5-1 | GCAATAGGAGCCTCTACATGAGCTT | Present study |
|  | IR5-1 | CATTTGTTATATTTTTTTCAGGAGT | Present study |
| 27 | IF5-2 | TATATAATTTGTATAACCGCAGATG | Present study |
|  | IR5-2 | TGAAAAGGAGCTACTCCTATTTTTA | Present study |

**Table S3. Primer sequences of *Leucochrysa pretiosa* mt genome used in this study.**

| Number | Primer ID | Nucleotide sequence (5’-3’) | Reference |
| --- | --- | --- | --- |
| 1 | F01(SPA-2756) | ACATTTTTTCCTCAACATTT | Simon *et al.* ^1^ |
|  | R01(SPA-3665) | CCACAAATTTCTGAACACTG | Simon *et al.* ^1^ |
| 2 | F03(SPA-3790) | CATTAAGTGACTGAAAGCAAGTA | Simon *et al.* ^1^ |
|  | R03(SPA-4552) | ATGACCTGCAATTATATTAGC | Simon *et al.* ^1^ |
| 3 | F03(SPA-3790) | CATTAAGTGACTGAAAGCAAGTA | Simon *et al.* ^1^ |
|  | R03(SPA-4552) | ATGACCTGCAATTATATTAGC | Simon *et al.* ^1^ |
| 4 | F06(SPA-5747) | CCATTTGAATGTGGRTTTGATCC | Simon *et al.* ^1^ |
|  | R06(SPA-6384) | AAAATTAAAAGCATAATATTGAAG | Simon *et al.* ^1^ |
| 5 | F12(SPB-11335) | CATATTCAACCAGAATGATA | Simon *et al.* ^1^ |
|  | R12(SPB-12067) | AATCGTTCTCCATTTGATTTTGC | Simon *et al.* ^1^ |
| 6 | F13(SPB-11876) | CGAGGTAAAGTACCACGTACTCA | Simon *et al.* ^1^ |
|  | R13(SPB-12595) | GTTGGATTTCTAACTTTATTRGARCG | Simon *et al.* ^1^ |
| 7 | F14(SPB-12261) | TACCTCATAAGAAATAGTTTGAGC | Simon *et al.* ^1^ |
|  | R14(SPB-13000) | TTACCTTAGGGATAACAGCGTAA | Simon *et al.* ^1^ |
| 8 | F15(SPB-12888) | CCGGTCTGAACTCAGATCATGTA | Simon *et al.* ^1^ |
|  | R15(SPB-13889) | ATTTATTGTACCTTTTGTATCAG | Simon *et al.* ^1^ |
| 9 | F17(SPB-14197) | GTAAAYCTACTTTGTTACGACTT | Simon *et al.* ^1^ |
|  | R17(SPB-14745) | GTGCCAGCAAYCGCGGTTATAC | Simon *et al.* ^1^ |
| 10 | F22(SPB-2756) | ACATTTTTTCCTCAACATTT | Simon *et al.* ^1^ |
|  | R22(SPB-3389) | TATTCATATCTTCAATATCATTGATG | Simon *et al.* ^1^ |
| 11 | F23(SPC-10621) | CTCATACTGATGAAATTTTGGTTC | Simon *et al.* ^1^ |
|  | R23(SPC-11526) | TTCTACTGGTCGTGCTCCAATTCA | Simon *et al.* ^1^ |
| 12 | TF3399 | ACAATTGGTCAYCAATGATAYTG | Simon *et al.* ^1^ |
|  | TR4061 | GAGAATAAGTTWGTTATCATTTTCA | Simon *et al.* ^1^ |
| 13 | TF413 | TTTGCCCATCTWGTWCCNCAAGG | Simon *et al.* ^1^ |
|  | TR4908 | CGAGTTAYATCTCGTCATCATTG | Simon *et al.* ^1^ |
| 14 | TF-J7572 | AAAGGGAATTTGAGCTCTTTTWGT | Simon *et al.* ^1^ |
|  | TR-N8487 | TATCAGSTAATATRGCWGCTCC | Simon *et al.* ^1^ |
| 15 | TF-J8941 | GAAACAGGAGCCTCAACATGWGC | Simon *et al.* ^1^ |
|  | TR-N9629 | GTTTGTGAGGGWGYTTTRGG | Simon *et al.* ^1^ |
| 16 | TF-J8641 | CCAGAAGAACATAANCCRTG | Simon *et al.* ^1^ |
|  | TR-N9629 | GTTTGTGAGGGWGYTTTRGG | Simon *et al.* ^1^ |
| 17 | TF210 | AATTAAGCTACTAGGTTCATACCC | Simon *et al.* ^1^ |
|  | TR1284 | ACARCTTTGAAGGYTAWTAGTTT | Simon *et al.* ^1^ |
| 18 | LF1 | ATAGAATCTCCTTTCACTATTGCAG | Present study |
|  | LR1 | ACTTTTTAGATGCAACCTAAATGTT | Present study |
| 19 | LF2 | GCAATTAGTTTCGACCTAATCTTTA | Present study |
|  | LR2 | ATTATAGAAAAGAATATATAGACGG | Present study |
| 20 | LF4 | TTTTCTTTTAAGGATATTGTTGGTA | Present study |
|  | LR4 | TATTACTATATTATGCTTTCAAAGC | Present study |
| 21 | LF5 | TATTTGGTCCTTTCGTACTAAAATA | Present study |
|  | LR5 | ATAATCATTTGTCTCTTAATTGGAG | Present study |
| 22 | LF6 | ATGGAAATTTCTAATCCTATATTTT | Present study |
|  | LR6 | TTGTATACCGTCGTTAACAGAATAT | Present study |
| 23 | LF2-2 | AACTTTGACCCCCTAAATATTCATT | Present study |
|  | LR2-2 | CCTGTATCTGCATTAGTACATTCAT | Present study |
| 24 | LF2-3 | TATCTTTGACACCACAAATCAATAT | Present study |
|  | LR2-3 | GATTACCTAAAGCTCATGTTGAAGC | Present study |
| 25 | LF2-4 | TCCATAAGATAATAAATCTATACCT | Present study |
|  | LR2-4 | GTTGCTCCTCAAAAAGATATTTGTC | Present study |
| 26 | LF3-1 | GAGGTTTACCTCCTTTTTTAGGATT | Present study |
|  | LR3-1 | CAGAATAACTATGTTCAGCAGGAGG | Present study |
| 27 | LF3-2 | AATCCTAAAGCACCTTCACATACTC | Present study |
|  | LR3-2 | GGTTGATTCATTAGTTTAATTGACG | Present study |
| 28 | LF4-1 | CATCTATAGTAGAAAGAGGAGCTGG | Present study |
|  | LR4-1 | CAACTCCTGTTAATCCTCCTACTGT | Present study |

**Table S4. Primer sequences of *Parankylopteryx* sp. mt genome used in this study.**

| Number | Primer ID | Nucleotide sequence (5’-3’) | Reference |
| --- | --- | --- | --- |
| 1 | F01(SPA-2756) | ACATTTTTTCCTCAACATTT | Simon *et al*. ^1^ |
|  | R01(SPA-3665) | CCACAAATTTCTGAACACTG | Simon *et al*. ^1^ |
| 2 | F10(SPA-8641) | CCAGAAGAACATAGCCCATG | Simon *et al*. ^1^ |
|  | R10(SPA-9629) | GTTTGTGAAGGTGTGTTGGG | Simon *et al*. ^1^ |
| 3 | F12(SPB-11335) | CATATTCAACCAGAATGATA | Simon *et* *al*. ^1^ |
|  | R12(SPB-12067) | AATCGTTCTCCATTTGATTTTGC | Simon *et al*. ^1^ |
| 4 | F13(SPB-11876) | CGAGGTAAAGTACCACGTACTCA | Simon *et al*. ^1^ |
|  | R13(SPB-12595) | GTTGGATTTCTAACTTTATTRGARCG | Simon *et al*. ^1^ |
| 5 | F14(SPB-12261) | TACCTCATAAGAAATAGTTTGAGC | Simon *et al*. ^1^ |
|  | R14(SPB-13000) | TTACCTTAGGGATAACAGCGTAA | Simon *et al*. ^1^ |
| 6 | F15(SPB-12888) | CCGGTCTGAACTCAGATCATGTA | Simon *et al*. ^1^ |
|  | R15(SPB-13889) | ATTTATTGTACCTTTTGTATCAG | Simon *et al*. ^1^ |
| 7 | F16(SPB-13342) | CCTTTGCACAGTCAAAATACTGC | Simon *et al*. ^1^ |
|  | R16(SPB-14220) | TTATGCACACATCGCCCGTC | Simon *et al*. ^1^ |
| 8 | F17(SPB-14197) | GTAAAYCTACTTTGTTACGACTT | Simon *et al*. ^1^ |
|  | R17(SPB-14745) | GTGCCAGCAAYCGCGGTTATAC | Simon *et al*. ^1^ |
| 9 | F20(SPB-586) | CCATTCCATTTYTGATTTCC | Simon *et al*. ^1^ |
|  | R20(SPB-1738) | TTTATTCGTGGAAATGCTATGTC | Simon *et al*. ^1^ |
| 10 | F22(SPB-2756) | ACATTTTTTCCTCAACATTT | Simon *et al*. ^1^ |
|  | R22(SPB-3389) | TATTCATATCTTCAATATCATTGATG | Simon *et al*. ^1^ |
| 11 | TF210 | AATTAAGCTACTAGGTTCATACCC | Simon *et al*. ^1^ |
|  | TR1284 | ACARCTTTGAAGGYTAWTAGTTT | Simon *et al*. ^1^ |
| 12 | TF-J7806 | GAMACAARACCTAACCCATCYCA | Simon *et al*. ^1^ |
|  | TR-N8727 | AAATCTTTRATTGCTTATTCWTC | Simon *et al*. ^1^ |
| 13 | TF413 | TTTGCCCATCTWGTWCCNCAAGG | Simon *et al*. ^1^ |
|  | TR510 | TCAACAAAATGTCARTAYCA | Simon *et al*. ^1^ |
| 14 | TF5470 | GCAGCTGCYTGATAYTGRCA | Simon *et al*. ^1^ |
|  | TR6384 | TATATTTAGAGYATRAYAYTGAAG | Simon *et al*. ^1^ |
| 15 | TF-J7572 | AAAGGGAATTTGAGCTCTTTTWGT | Simon *et al*. ^1^ |
|  | TR-N8487 | TATCAGSTAATATRGCWGCTCC | Simon *et al*. ^1^ |
| 16 | TF-J9648 | ACCTAAAGCTCCCTCACAWAC | Simon *et al*. ^1^ |
|  | TR-N10608 | CCAAGTARTGAWCCAAARTTTCA | Simon *et al.* ^1^ |
| 17 | PF1 | ATTGGTAAAAGAGATTTTATCTCAT | Present study |
|  | PR1 | TCATGTTGCCATTATAATAATTCTA | Present study |
| 18 | PF2 | CATGATCAAAAATTTCTTTACAAAA | Present study |
|  | PR2 | CAGCCATCTAATGAAGATTTATTTA | Present study |
| 19 | PF3 | AAATTTAGTACTTTTTGATTCCACA | Present study |
|  | PR3 | AATTATCATATTGATGGAATCATTT | Present study |
| 20 | PF5 | TATAATCATAATATACATAATTCTC | Present study |
|  | PR5 | TTTGAAGGTAGTTTAATTCCTACAT | Present study |
| 21 | PF7 | CACCTACTAAAACACAAATTACAAT | Present study |
|  | PR7 | TCTTTTAATTGAAGGCTAGAATGAA | Present study |
| 22 | PF8 | AACCCTGATACAAAAGGTACATTAA | Present study |
|  | PR8 | TATTCTCGAAACTTAAAGAATTTGG | Present study |
| 23 | PF2-1 | GCAGGTATTTCATCAATTTTAGGGG | Present study |
|  | PR2-1 | CAGAATATCTATGTTCAGCTGGAGG | Present study |
| 24 | PF2-2 | TCATCAGGAGTAACAATTACTTGAG | Present study |
|  | PR2-2 | AATGCTCCTTGATTTCATTCATGAT | Present study |
| 25 | PF2-3 | TGAAACCAAAATAGAGGTATATCAC | Present study |
|  | PR2-3 | AGGTTGAGATGGATTAGGTTTAGTT | Present study |
| 26 | PF2-4 | ACGATTAATTGATTCACTAGCAGTT | Present study |
|  | PR2-4 | TCTACTGGTCGAGCTCCAATTCAAG | Present study |
| 27 | PF3-1 | CCAGATATAGCATTCCCTCGAATAA | Present study |
|  | PR3-1 | CGAGTATCTACATCTATACC | Present study |
| 28 | PF3-2 | CTTCATGGAACTCAATTTAC | Present study |
|  | PR3-2 | GGGAGAAGCTCTATTTTGTAAAGAA | Present study |
| 29 | PF3-3 | ACAATTAGTTTCGACCTAATCTTAG | Present study |
|  | PR3-3 | GCTGGGTTAGGAGCAAATTATGAAT | Present study |
| 30 | PF3-4 | GCTCCTAACCCAGCTATAAATATTG | Present study |
|  | PR3-4 | GATTTGTGGTGTCAAAGATATGAGG | Present study |
| 31 | PF3-6 | ATCGTGGATTATCAATTATAGAACA | Present study |
|  | PR3-6 | GAAAGGGGCAATTCCTATTTTTAAA | Present study |
| 32 | PF5-1 | AATAAATATACTCCTGCTTGTAAAC | Present study |
|  | PR5-1 | TACTATTGCTGCTACAATAAAAGGT | Present study |
| 33 | PF6-1 | TATTTACATACTGGACGAGG | Present study |
|  | PR6-1 | GATAAAACTAATGCAATTACTCCTC | Present study |

**Table S5. Partitions and partition-specific models for BI analysis estimated with PartitionFinder.**

| Partition | Positions included | Model for BI |
| --- | --- | --- |
| 1 | 12S, ATP6_pos1, ATP6_pos2, ATP6_pos3, ATP8_pos1, ATP8_pos2, ATP8_pos3, COX1_pos2, COX1_pos3, ND3_pos1, ND4L_pos2, ND4_pos2, ND5_pos2, ND6_pos2 | GTR+I+G |
| 2 | 16S,COX1_pos1, ND4_pos1, ND4_pos3 | GTR+I+G |
| 3 | COX2_pos1, COX3_pos1, ND1_pos1, ND2_pos1, ND4L_pos1,cytB_pos1 | HKY+G |
| 4 | COX2_pos2, COX3_pos2, ND1_pos2, ND2_pos2, cytB_pos2 | GTR+I+G |
| 5 | COX2_pos3, COX3_pos3, ND1_pos3, ND2_pos3, ND3_pos2, ND4L_pos3, ND5_pos3, ND6_pos3, cytB_pos3 | GTR+I+G |
| 6 | ND3_pos3, ND5_pos1, ND6_pos1 | GTR+I+G |

**Table S6. Partitions and partition-specific models for ML analysis estimated with PartitionFinder.**

| Partition | Positions included | Model for BI |
| --- | --- | --- |
| 1 | 12S, ATP6_pos1, ATP6_pos2, ATP6_pos3, ATP8_pos1, ATP8_pos2, ATP8_pos3,COX1_pos2, COX1_pos3,  ND4L_pos2, ND4_pos2, ND5_pos2, | GTR+I+G |
| 2 | 16S,COX1_pos1, ND4_pos1, ND4_pos3 | GTR+I+G |
| 3 | COX2_pos1, COX3_pos1, ND1_pos1, ND2_pos1, ND4L_pos1,cytB_pos1 | GTR+G |
| 4 | COX2_pos2, COX3_pos2, ND1_pos2, ND2_pos2, cytB_pos2 | GTR+I+G |
| 5 | COX2_pos3, COX3_pos3, ND1_pos3, ND2_pos3, ND3_pos 1,ND3_pos2, ND4L_pos3, ND5_pos3, ND6_pos 2,ND6_pos3, cytB_pos3 | GTR+I+G |
| 6 | ND3_pos3, ND5_pos1, ND6_pos1 | GTR+I+G |

**Reference**

1. Simon, C., Buckley, T. R., Frati, F., Stewart, J. B. & Beckenbach, A. T. Incorporating molecular evolution into phylogenetic analysis, and a new compilation of conserved polymerase chain reaction primers for animal mitochondrial DNA. *Ann. Rev. Ecol. Evol. Syst.* **37**, 545–579 (2006).
